# Phase II randomized trial of capecitabine with bevacizumab and external beam radiation therapy as preoperative treatment for patients with resectable locally advanced rectal adenocarcinoma: long term results

**DOI:** 10.1186/s12885-020-07661-z

**Published:** 2020-11-27

**Authors:** Ramón Salazar, Jaume Capdevila, Jose Luis Manzano, Carles Pericay, Mercedes Martínez-Villacampa, Carlos López, Ferrán Losa, María José Safont, Auxiliadora Gómez-España, Vicente Alonso-Orduña, Pilar Escudero, Javier Gallego, Beatriz García-Paredes, Amalia Palacios, Sebastiano Biondo, Cristina Grávalos, Enrique Aranda, on behalf of the Spanish Cooperative Group for the Treatment of Digestive Tumors (TTD)

**Affiliations:** 1 Medical Oncology. Oncobell Program IDIBELL Institut Català d’Oncologia Hospital Duran i Reynals, CIBERONC, Barcelona, Spain; 2 Medical Oncology, Hospital Universitari Vall d’Hebrón, Barcelona, Spain; 3 Medical Oncology, ICO. Hospital Germans Trias i Pujol, Barcelona, Spain; 4 Medical Oncology, C. S. Parc Taulí, Barcelona, Spain; 5 Hospital Marqués de Valdecilla, Santander, Spain; 6 Medical Oncology, Hospital Sant Joan Despí - Moisés Broggi, Barcelona, Spain; 7 Medical Oncology, Hospital General Universitario, Valencia, Spain; 8 Medical Oncology. IMIBIC, Hospital Reina Sofía, CIBERONC Instituto de Salud Carlos III, University of Córdoba, Córdoba, Spain; 9 Medical Oncology. Hospital Miguel Servet, Zaragoza, Spain; 10 Medical Oncology. Hospital C. Universitario Lozano Blesa, Zaragoza, Spain; 11 Medical Oncology. Hospital General Universitario de Elche, Alicante, Spain; 12 Medical Oncology, Hospital Clínico San Carlos, Instituto de Investigación Hospital Clinico San Carlos (IdISSC), CIBERONC, Madrid, Spain; 13 Radiation Oncology, Hospital Reina Sofía, Córdoba, Spain; 14 General and Digestive Surgery. Hospital Universitario de Bellvitge, Barcelona, Spain; 15 Medical Oncology. Hospital 12 de Octubre, Madrid, Spain

**Keywords:** Locally-advanced rectal cancer, Bevacizumab, Neoadjuvant, Chemoradiotherapy

## Abstract

**Background:**

Preoperative chemoradiotherapy with capecitabine is considered as a standard of care for locally advanced rectal cancer. The “Tratamiento de Tumores Digestivos” group (TTD) previously reported in a randomized Ph II study that the addition of Bevacizumab to capecitabine-RT conferred no differences in the pre-defined efficacy endpoint (pathological complete response).

We present the follow-up results of progression-free survival, distant relapse-free survival, and overall survival data at 3 and 5 years.

**Methods:**

Patients (pts) were randomized to receive 5 weeks of radiotherapy (45 Gy/25 fractions) with concurrent Capecitabine 825 mg/m2 twice daily, 5 days per week with (arm A) or without (arm b) bevacizumab (5 mg/kg once every 2 weeks).

**Results:**

In our study, the addition of bevacizumab to capecitabine and radiotherapy in the neoadjuvant setting shows no differences in pathological complete response (15.9% vs 10.9%), distant relapse-free survival (81.0 vs 80.4 and 76.2% vs 78.2% at 3 and 5 years respectively), disease-free survival (75% vs 71.7 and 68.1% vs 69.57% at 3 and 5 years respectively) nor overall survival at 5-years of follow-up (81.8% vs 86.9%).

**Conclusions:**

the addition of bevacizumab to capecitabine plus radiotherapy does not confer statistically significant advantages neither in distant relapse-free survival nor in disease-free survival nor in Overall Survival in the short or long term.

**Trial registration:**

EudraCT number: 2009–010192-24.

Clinicaltrials.gov number: NCT01043484.

**Supplementary Information:**

The online version contains supplementary material available at 10.1186/s12885-020-07661-z.

## Background

Significant progress in the management of locally advanced rectal cancer (LARC) has been achieved during the last two decades. This includes the use of postoperative Radiotherapy [1]; postoperative chemoradiotherapy (CRT) [2]; and the widespread implementation of total mesorectal excision and autonomic nerve preservation [3]. The German Rectal Cancer Study Group established that there was a significant improvement in local control, toxicity profile and sphincter preservation in patients with LARC treated with preoperative versus postoperative CRT [4]. Currently perioperative CRT is worldwide accepted as standard treatment in patients with locally advanced (T3-T4) rectal cancer because it improves local control and survival. This strategy provides early exposure to systemic therapy, maximizes downstaging, and increases the options of sphincter-sparing surgery.

Although local recurrences with this approach have dropped from 20 to 40% [5] to less than 10% with CRT [6], the impact of 5FU added to radiotherapy in survival has been questioned as systemic treatment following the results of Bujko et al [7]. Probably a fluoropyrimidine-alone chemotherapy regimen is unlikely to be adequate as systemic treatment because its modest single-agent activity in colorectal cancer might be compromised by the reduced dosing necessary for safe concurrent administration with radiotherapy.

Capecitabine is a drug designed to improve the convenience of 5FU and has largely replaced it in metastatic and localized disease along with radiotherapy [8].

In the metastatic setting, bevacizumab (BVZ) increases the activity of polychemotherapy [9] and fluoropyrimidine monotherapy [10, 11] although its activity in combination with FOLFOX in the first-line setting has been questioned [12]. The advantage conferred by the treatment with BVZ plus chemotherapy in the treatment of CRC cancer patients could be due to increased tumour cell sensitivity to the action of the chemotherapy, or the better distribution of chemotherapy into the tumour. It is an attractive drug to be used in combination with fluoropyrimidines and radiation therapy given its good tolerance, especially in a population whose average age is over 65 years. Therefore, we published a randomized phase II study in 2015 [13] and we present here its long term results in survival outcomes.

## Methods

### Trial design

This study was a national, multicenter, open-label randomized phase II trial performed by the Spanish Cooperative Group for the Treatment of Digestive Tumors (TTD Group). The study was performed following the Declaration of Helsinki and Good Clinical Practice Guidelines, and written informed consent was obtained from all patients before taking part in the study. The Reference Ethics Committee was Comité Ético de Investigación Clínica del Hospital Universitario 12 de Octubre, Avda de Córdoba, s/n, 28,041 Madrid. The protocol was locally approved in all the participating centers by the institutional review boards. This study adhered to CONSORT guidelines and has strictly followed CONSORT recommendations.

The main objective of the study was the complete pathological response (ypCR) rate, defined as ypT0 and ypN0 in the surgical specimen and was previously reported as well as the possible molecular dynamic and predictive factors of response in tissue of the diagnostic tumoral biopsy and the angiogenic profile. Other secondary objectives were: Disease-free survival (DFS) and distant relapse-free survival (DRFS) at 3 and 5 years, and overall survival (OS).

Disease-free survival (DFS) was defined as the time elapsed between the randomization and the time of the first local relapse; distant relapse-free survival (DRFS) was defined as the time from randomization until the moment of first distant relapse. OS was defined as the time elapsed between randomization and date of death.

Once it was corroborated that patients had signed the informed consent and fulfilled selection criteria, they were randomly assigned in a 1:1 ratio to CRT treatment with or without BVZ. Randomization was performed centrally, by PIVOTAL Contract Research Organization.

### Patient selection

Eligibility criteria included patients with clinical stages II and III LARC within < 15 cm from the anal verge (T and N defined by pelvic magnetic resonance imaging (MRI) and categorized according to the AJC on Cancer Staging Manual 6th Edition), candidates to surgical resection, with adequate organ and bone marrow function, ECOG performance status 0 or 1, age ≥ 18 years and chemotherapy and radiation therapy naïve. Other patient’s selection criteria previously described [13].

### Treatment schedule

Patients were randomly assigned to radiotherapy (45 Gy delivered in 25 daily fractions over 5 weeks) with capecitabine or capecitabine plus BVZ and stratified by center and tumour location in upper, middle and lower rectum. Arm A consisted of BVZ (5 mg/kg) on day 1 of weeks 1, 3, and 5 plus capecitabine and patients included in arm B received only capecitabine as previously described [13]. Patients underwent surgery 6–8 weeks after the completion of CRT as part of clinical practice. The type of postoperative chemotherapy was not defined, nor if it should be used.

Capecitabine dose-modification criteria were established but for bevacizumab no dose-reductions were contemplated.

### Evaluations during the study

A complete colonoscopy with biopsy, pelvic MRI, thoracoabdominal computed tomography (or abdominal computed plus tomography thoracic x-ray), and electrocardiogram were performed prior to the beginning of the study. Laboratory studies (haematology, chemistry, coagulation profile, urinalysis, carcinoembryonic antigen) were repeated before the start of each treatment cycle. Histologic assessment (ypT, ypN) and grading of regression (according to Mandard scale) were assessed after surgery. A follow-up of up to five years was planned after surgery.

### Sample size calculation and statistical analysis

The sample size and the decision rule were based on the Simon, Wittes, and Ellenberg (SWE) method for randomized phase II trials. Forty-one patients were needed per arm assuming an ypCR proportion of 15% in one of the arms, a difference between arms of 10%, and accepting a probability of making a correct selection of 87%. This number was increased by 10% until 90 considering the possible loss of evaluable patients. All statistical tests were two-sided. Additional data have been previously published [13].

All eligible and consenting patients (the full analysis population) were included in the analyses of OS, DRFS, and DFS and the cumulative incidence rates of local and distant recurrences, according to the intention-to-treat principle.

Univariate analyses of survival were carried out by the Kaplan–Meier method, and the evaluation of differences was performed with the log-rank test.

## Results

We previously published the baseline characteristics of the patients participating in this study [13]; here we present the long-term follow-up results at 3 and 5 years of DFS, DRFS, and OS.

Ninety patients were included from December 2009 until March 2011 in 12 hospitals in Spain; 44 were randomly assigned to arm A and 46 to arm B.

Patients received a median of 3 (range 2–3) BVZ cycles, one dose delay of BVZ, there were no cases of BVZ dose reductions or discontinuations. For both arms, the median number of capecitabine received cycles was 3 (range 1–3). There were not any statistically significant differences regarding the number of patients with delayed capecitabine cycles, occurring in 66.67% cycles in arm A and 77.78 in arm B. Only 5 patients had early treatment discontinuation (2 in arm A, 3 in arm B); 1 patient required a capecitabine dose reduction.

Overall, the median dose intensity was equivalent to 85% of expected, observing similar values across both groups.

### Safety, treatment-related toxicity and surgical outcomes

*Treatment was well tolerated*. Eighty patients (88.89%) reported treatment-emergent adverse events. *There were no differences in grade 3–4 treatment-related toxicity.* No grade 3 or greater haematological toxicity *was reported*. Two patients in arm A presented hypertension. One patient in the study received only 2 cycles of BVZ due to capecitabine-related toxicity.

Surgery was delayed more than 9 weeks after the end of treatment in 4 patients in arm A, and 5 in arm B. There were no differences in the surgical technique performed nor in the frequency of anal sphincter preservation. Nineteen patients (43%) and 18 (39%) patients in arm A and B experienced at least one postoperative complication, respectively. 10 patients (7 in arm A (16.3%) and 3 in arm B (6.5%)) required repeat surgery, due to suture failures (Table 1). There were no perioperative deaths. There were no differences for hospitalization time among arms: 11 vs 10 days for arm A and b respectively.

**Table 1:** Post operatory toxicities

	ARM A		ARM B	
	Grade 3 (%)	Grade 4 (%)	Grade 3 (%)	Grade 4 (%)
**Abdominal abscess**	2 (4.55)	–	–	–
**Peritonitis**	2(4.55)	–	–	–
**Bacteremia**	1(2.27)	–	–	–
**Perineal abscess**	–	–	1(2.17)	–
**Septic shock**	–	1(2.27)	–	–
**Tracheobronchitis**	1(2.27)	–	–	–
**Urinary tract infection**	1(2.27)	–	–	–
**Wound infection**	1(2.27)	–	–	–
**Pelvic abscess**	–	–	1(2.17)	–
**Post-operatory wound infection**	–	–	1(2.17)	–
**Intestinal obstruction**	2(4.55)	–	–	–
**Proctitis**	1(2.27)	–	–	–
**Enterovesical fistula**	–	–	–	1(2.17)
**Intestinal ischemia**	–	–	1(2.17)	–
**Paralytic ileus**	–	–	1(2.17)	–
**Anastomotic failure**	–	1(2.27)	–	1(2.17)
**Complication of a gastrointestinal stoma**	1(2.27)	–	–	..
**Wound dehiscence**	.	1(2.27)	–	–
**Coagulopathy**	1 (2.27)	–	–	–
**Anemia**	–	–	1(2.17)	–
**Iron deficiency anemia**	–	–	1(2.17)	–
**Auricular fibrillation**	1 (2.27)	–	–	–
**Cardiorespiratory arrest**	.	1(2.27)	–	–
**Acute renal injury**	1 (2.27)	–	–	–
**Renal failure**	–	–	1(2.17)	–
**Vaginal fistula**	–	–	1(2.17)	–
**Hepatic insufficiency**	1 (2.27)	–	–	–
**Chest pain**	1 (2.27)	–	–	–
**Blood calcium**	1 (2.27)	–	–	–
**Fistula**	–	1(2.27)	–	–
**Hypertension**	–	–	1(2.17)	–

Eighty-three percent of the patients (34 patients in arm A and 41 patients in arm B) received adjuvant treatment, and 20% (8 patients in arm A and 10 in arm B) received further systemic treatment for metastatic disease.

### Response to treatment

Pathological complete response was achieved in 15.9% (95% CI 7–31%) and 10.9% (95% CI 4–24%) in arms A and B, respectively. Although there were no differences in histologic tumoral regression, a statistic trend was found in primary tumour pathological downstaging (Table 2).

**Table 2:** Tumoral regression among 89 resected patients

		A (BVZ + CAPE + RT) (***N*** = 44)	B (CAPE + RT) (***N*** = 46)	Total (***N*** = 90)	***P*** Value Test
Pathologic response per tumoral regression (TRG)*					
TRG 1(Complete pathologic response)	*N*(%)	8 (18.18)	5 (10.87)	13 (14.44)	Fisher: 0.1458
TRG 2	*N*(%)	8 (18.18)	15 (32.61)	23 (25.56)	
TRG 3	*N*(%)	14 (31.82)	19 (41.30)	33 (36.67)	
TRG 4	*N*(%)	12 (27.27)	6 (13.04)	18 (20.00)	
TRG 5(Disease progression)	*N*(%)	0 (0.00)	1 (2.17)	1 (1.11)	
ND	*N*(%)	1 (2.27)	0 (0.00)	1 (1.11)	
Missing	*N*(%)	1 (2.27)	0 (0.00)	1 (1.11)	
**ypT improvement**					
Better	*N*(%)	26 (59.09)	18 (39.13)	44 (48.89)	**Fisher: 0.0429**
Remained the same	*N*(%)	16 (36.36)	28 (60.87)	44 (48.89)	
Worse	*N*(%)	1 (2.27)	0 (0.00)	1 (1.11)	
Missing	*N*(%)	1 (2.27)	0 (0.00)	1 (1.11)	
**ypN improvement**					
Better	*N*(%)	24 (54.55)	35 (76.09)	59 (65.56)	Fisher: 0.0865
Remained the same	*N*(%)	15 (34.09)	8 (17.39)	23 (25.56)	
Worse	*N*(%)	4 (9.09)	2 (4.35)	6 (6.67)	
No evaluable*	*N*(%)	0 (0.00)	1 (2.17)	1 (1.11)	
Missing	*N*(%)	1 (2.27)	0 (0.00)	1 (1.11)	
**ypT and ypN improvement**					
Improvement in both	*N*(%)	18 (40.91)	16 (34.78)	34 (37.78)	Fisher: 0.5612
Improvement in one	*N*(%)	14 (31.82)	20 (43.48)	34 (37.78)	
No improvement	*N*(%)	11 (25.00)	9 (19.57)	20 (22.22)	
Non-evaluable*	*N*(%)	0 (0.00)	1 (2.17)	1 (1.11)	
Missing	*N*(%)	1 (2.27)	0 (0.00)	1 (1.11)	

* A patient was reported as NX

### Treatment after surgery

Thirty-four patients in arm A and 41 in arm B received adjuvant treatment. Seventeen cases from arm A received combination with oxaliplatin compared with 24 in arm B (Table 3).

**Table 3:** Adjuvant treatment after surgery

		A (BVZ + CAPE + RT) (***N*** = 44)	B (CAPE + RT) (***N*** = 46)	Total (***N*** = 90)
**Patients receiving adjuvant chemotherapy**				
No	*n* (%)	10 (22.73)	5 (10.87)	15 (16.67)
Yes	*n* (%)	34 (77.27)	41 (89.13)	75 (83.33)
Adjuvant schedule				
capecitabine	*n* (%)	18 (40.9)	21 (45.65)	39 (43.33)
XELOX	*n* (%)	10 (22.73)	15 (32.61)	25 (27.78
FOLFOX	*n* (%)	6 (13.64)	8 (17.39)	14 (15.56)
Oxaliplatin + Raltitrexed	*n* (%)	1 (2.27)	1 (2.17	2 (2.22)
5-Fu	*n* (%)	–	2 (4.34)	2 (2.22)

### Disease-free survival (DFS), distant-relapse free survival (DRFS) and overall survival (OS)

**Fig. 1:**
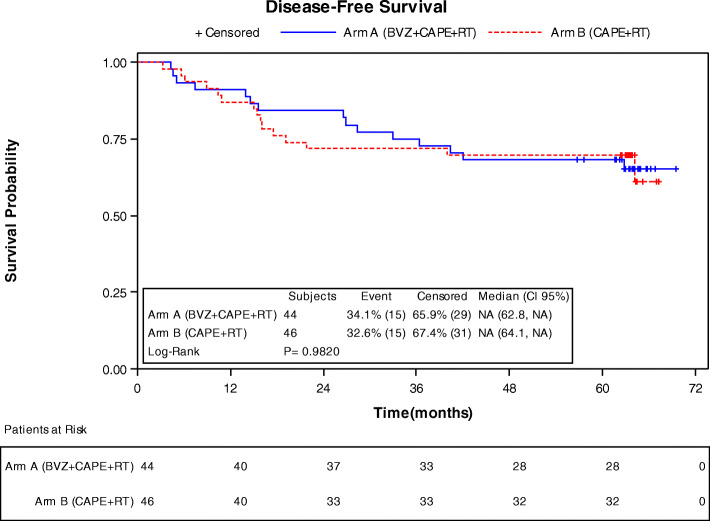
Disease-free survival

**Fig. 2:**
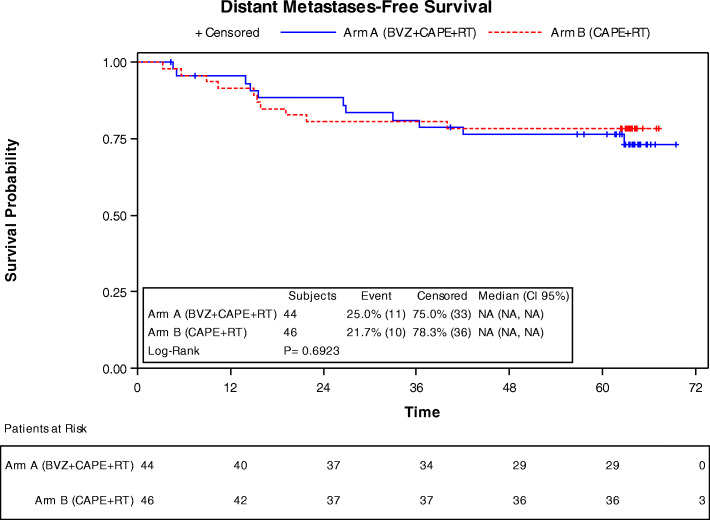
Distant relapse-free survival

DFS at 3 years in arm A (Fig. 1) was of 75% (CI 95% 59.42, 85.30); and 71.7% (CI95% 56.3, 82.50) in arm B. At 5 years the DFS probability was of 68.18 (CI95% 52.27, 79.76) and 69.57% (CI95% 54.09, 80.71) for arms A and B, respectively. DRFS probability at 3 and 5 years was of 81.05% (65.65, 90.04) and 76.2% (60.27, 86.43) for arm A, and 80.43% (65.77, 89.30) and 78.26% (63.36, 87.66) for Arm B (Table 4). Eleven patients (25%) of arm A and 10 patients (21.7%) of arm B had a distant relapse, (Fig. 2).

**Table 4:** Distant relapse-free survival and progression free survival

	Arm A (BVZ + CAPE + RT)	Arm B (CAPE + RT)
**Distant relapse-free survival**	**44 patients**	**46 patients**
No of patients with event	11 (25.00%)	10 (21.74%)
No of censored patients	33 (75.00%)	36 (78.26%)
**Percent Survival (%, 95 CI)**		
36 Time (months)	81.05 (65.65, 90.04)	80.43 (65.77, 89.30)
60 Time (months)	76.20 (60.27, 86.43)	78.26 (63.36, 87.66)
**Kaplan-Meier model**		
*P*-value (Log-rank)		0.6923
**Cox Model**	**Hazard ratio (95% CI)**	**Cox Model** ***P*-value**
Arm A (BVZ + CAPE+RT) vs Arm B (CAPE+RT)	1.1887 (0.5047, 2.8000)	0.6924
**Disease free survival**		
No of patients with event	15 (34.09%)	15 (32.61%)
Earliest contributing event:		
Distant metastases	11	10
Second tumor	1	4
Death	3	1
No of censored patients	29 (65.91%)	31 (67.39%)
**Lab**		
Median (95% CI)	NA (62.76, NA)	NA (64.13, NA)
25th–75th percentile	34.67 - NA	19.22 - NA
**Percent Survival (%, 95 CI)**		
36 months	75.00 (59.42, 85.30)	71.74 (56.36, 82.50)
60 months	68.18 (52.27, 79.76)	69.57 (54.09, 80.71)
**Kaplan-Meier model**		
*P*-value (Log-rank)		0.9820
**Cox Model**	**Hazard ratio (95% CI)**	**Cox Model** ***P*-value**
Arm A (BVZ + CAPE+RT) vs Arm B (CAPE+RT)	1.0083 (0.4927, 2.0635)	0.9820

In an exploratory analysis, we observed a benefit in DFS for those patients receiving BVZ that achieved a T-downstaging (*p* = 0.012), as well as for those receiving BVZ obtaining an N-downstaging (*p* = 0.005). supplementary Figs. 1 and 2.

Seventeen patients died during the study. In arm A, one patient died from advanced dementia, another from complications of chronic obstructive pulmonary disease, and 8 from disease progression. In arm B, 2 (38.6%) patients died from unknown causes, 1 (14.3%) form respiratory insufficiency, and 4 (57.4%) from disease progression.

**Fig. 3:**
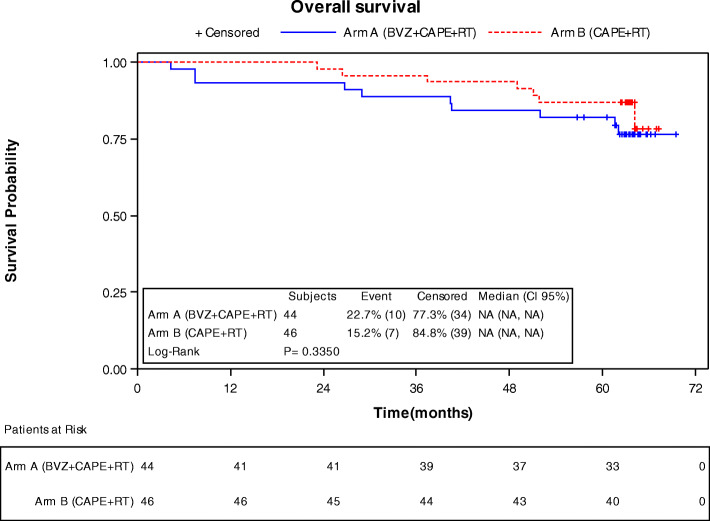
Overall survival

OS probability at 3 and 5 years was of 88.6% (CI 95% 74.83, 95.11) and 81.82 (CI95% 66.92, 90.46) for arm A, and 95.65% (CI95% 83.71, 98.89) and 86.96 (CI95% 73.25, 93.92) for Arm B (Table 5). There were no OS statistically significant differences among both treatment arms (*p* = 0.33 Fig. 3).

**Table 5:** Overall survival

	Arm A (BVZ + CAPE + RT)	Arm B (CAPE + RT)
**Summary of events**		
No of patients	44	46
No of patients with event	10 (22.73%)	7 (15.22%)
No of censored patients	34 (77.27%)	39 (84.78%)
**Lab**		
Median (95% CI)	NA (NA, NA)	NA (NA, NA)
25th–75th percentile	NA - NA	NA - NA
**Percent Survival (%, 95 CI)**		
36 months	88.64 (74.83, 95.11)	95.65 (83.71, 98.89)
60 months	81.82 (66.92, 90.46)	86.96 (73.25, 93.92)
**Kaplan-Meier model**		
*P*-value (Log-rank)		0.3350
**Cox Model**	**Hazard ratio (95% CI)**	**Cox Model** ***P*-value**
Arm A (BVZ + CAPE+RT) vs Arm B (CAPE+RT)	1.6013 (0.6091, 4.2097)	0.3397

## Discussion

At present, LARC treatment has reached an elevated percentage of local control with optimization of surgery and neoadjuvant radiotherapy or chemoradiotherapy; the current efforts are aimed at improving quality of life in low-risk cases, and both increasing local control and reducing distant metastases that are the leading cause of neoplastic death in these patients [14].

In the last decade, most drugs tested concomitantly with radiotherapy plus fluoropyrimidines in the preoperative setting have not succeeded to become new treatment standards despite having previously shown efficacy in the metastatic colorectal cancer setting. These included oxaliplatin [15], irinotecan [16] and monoclonal antibodies (MoAb) such as BVZ and Cetuximab [17]. The most promising exploratory combinations involved oxaliplatin, that has been intensively studied in phase II trials, with increased ypCR rate up to 21–37% [18, 19] but no benefit in long term survival outcomes [20–24].

Due to the discouraging results of irinotecan in the adjuvant colon cancer setting, this drug has not been extensively studied as neoadjuvant treatment. The ypCR rate communicated for Wang et al is in the rage of those reported with oxaliplatin combinations, although long term results have not been reported [16].

The main objective of our study, ypCR (T and N), did not show differences between treatment arms and it is in the range of other studies (8–27%) [25–27], and although we found an statistical trend to yPT downstaging with capecitabine plus bevacizumab this was an unplanned exploratory finding. This is in line with other phases I and II trials of BVZ in combination with RT and fluoropyrimidines alone (5-Fluorouracil [28] or capecitabine [29]) or in combination with oxaliplatin [18, 30–33] with response rates not better than with chemotherapy alone (13–18%). Now we report the results of long term DFS, DRFS, and OS, where we have found no significant differences between treatment arms. Interestingly 5-year DFS and OS results observed in our study were somewhat inferior to others, which probably relates to the randomized design and less restricted selection criteria in our study. Toxicities observed in this study were not different between arms; nevertheless, more patients required surgery due to suture failures. This might be related to BVZ despite the long time elapsed between bevacizumab’s last dose and surgery.

Some differences in the number of patients that received postoperative chemotherapy and regimens used were observed. Oxaliplatin was used in 41 patients based on the results of adjuvant colon cancer clinical trials [34, 35]. This drug has been studied in rectal cancer in association with 5FU in several randomized trials in rectal cancer showing no improvement in survival [36–38] except for the ADORE trial in which patients were randomized after surgery [39]. In this study, the addition of OXL to adjuvant 5FU in patients with positive lymph nodes after surgery improved OS. However, the role of adjuvant chemotherapy for patients with rectal cancer remains controversial, and two Pooled analyses have shown conflicting results [40, 41]. Furthermore, recommendations of complementary treatment of rectal cancer patients are mostly based on the results of old trials (before pre-operative treatment was standard) [2, 42, 43]. We therefore assume that differences in the adjuvant treatment have not affected substantially the long-term results of our study. OS survival and DFS results from our study are consistent with other randomized studies recently reported [19, 20, 32, 38, 44].

## Conclusion

No differences in OS, DFS, or DRFS were obtained with the inclusion of bevacizumab in the preoperative setting of rectal cancer. These results are in line with those of the main endpoint of the study and other reports that have shown that the addition of bevacizumab does not improve yPCR. At this time treatment with fluoropyrimidines plus radiotherapy should continue to be considered the standard neoadjuvant treatment in rectal cancer.

## Supplementary Information

**Additional file 1:** **Supplementary Figure 1.** DFS – T-downstaging and treatment arm

**Additional file 2:** **Supplementary Figure 2.** DFS. N-downstaging and treatment arm.

## Data Availability

The datasets analyzed during the current study are available from the corresponding author on reasonable request.

## Abbreviations

AJC: American Joint Committee
CRT: Chemoradiotherapy
ypCR: Complete pathologic response
DFS: Disease-free survival
DRFS: Distant relapse-free survival
EBRT: External Beam Radiation Therapy
ECOG: Eastern Cooperative Oncology Group
EORTC: European Organization for Research and Treatment of Cancer
5-FU: 5-Fluorouracil
BVZ: Bevacizumab
LARC: Locally advanced rectal carcinoma
MoAb: Monoclonal antibodies
MRI: Magnetic resonance imaging
NCI-CTC: National Cancer Institute Common Toxicity Criteria
OS: Overall Survival
RT: Radiotherapy
SWE: Simon, Wittes, and Ellenberg
TRG1: Primary tumor regression
TME: Total mesorectal excision
TTD: Treatment of Digestive Tumors
VEGF: Vascular endothelial growth factor

## References

[1] Gastrointestinal Tumor Study Group (1985). Prolongation of the disease-free interval in surgical treated rectal carcinoma. N Engl J Med.

[2] Krook JE, Moertel CG, Gunderson LL, Wieand HS, Collins RT, Beart RW (1991). Effective surgical adjuvant therapy for high-risk rectal carcinoma. N Engl J Med.

[3] Havenga K, Enker WE, Norstein J, Moriya Y, Heald RJ, van Houwelingen, et al. (1999). Improved survival and local control after total mesorectal excision or D3 lymphadenectomy in the treatment of primary rectal cancer: an international analysis of 1411 patients. Eur J Surg Oncol.

[4] Sauer R, Becker H, Hohenberger W, Rödel C, Wittekind C, Fietkau R (2004). Preoperative versus postoperative Chemoradiotherapy for rectal Cancer. N Engl J Med.

[5] Havenga K, Enker WE, Norstein J (1999). Improved survival and local control after total mesorectal excision or D3 lymphadenectomy in the treatment of primary rectal cancer: an international analysis of 1411 patients. Eur J Surg Oncol.

[6] Bosset JF, Collette L, Calais G (2006). Chemotherapy with preoperative radiotherapy in rectal cancer. N Engl J Med.

[7] Bujko K, Nowacki MP, Nasierowska-Guttmejer A, Michalski W, Bebenek M, Kryj M (2006). Long-term results of a randomized trial comparing preoperative short-course radiotherapy with preoperative conventionally fractionated chemoradiation for rectal cancer. Br J Surg.

[8] Hofheinz R-D, Wenz F, Post S, Matzdorff A, Laechelt S, Hartmann JT (2012). Chemoradiotherapy with capecitabine versus fluorouracil for locally advanced rectal cancer: a randomised, multicentre, non-inferiority, phase 3 trial. Lancet Oncol.

[9] Herbert Hurwitz H, Fehrenbacher L, Novotny W, Cartwright T, Hainsworth J, Heim W (2004). Bevacizumab plus Irinotecan, fluorouracil, and Leucovorin for metastatic colorectal Cancer. N Engl J Med.

[10] Cunningham D, Lang I, Marcuello E, Lorusso V, Ocvirk J, Shin DB (2013). Bevacizumab plus capecitabine versus capecitabine alone in elderly patients with previously untreated metastatic colorectal cancer (AVEX): an open-label, randomized phase 3 trial. Lancet Oncol.

[11] Kabbinavar FF, Schulz J, McCleod M, Patel T, Hamm JT, Hecht JR (2005). Addition of Bevacizumab to bolus fluorouracil and Leucovorin in first-line metastatic colorectal Cancer: results of a randomized phase II trial. J Clin Oncol.

[12] Saltz LB, Clarke S, Diaz-Rubio E, Scheithauer W, Figer A, Wong R (2008). Bevacizumab in Combination With Oxaliplatin-Based Chemotherapy As First-Line Therapy in Metastatic Colorectal Cancer: A Randomized Phase III Study. J Clin Oncol.

[13] Salazar R, Capdevila J, Laquente B, Manzano JL, Pericay C, Martínez Villacampa M, et al. A randomized phase II study of capecitabine-based chemoradiation with or without bevacizumab in resectable locally advanced rectal cancer: clinical and biological features. BMC Cancer [Internet] 2015 Feb [Cited 2015 Feb 26; 15: 60]. Available from https://www.ncbi.nlm.nih.gov/pmc/articles/PMC4343271/ DOI: 10.1186/s12885-015-1053-z. 10.1186/s12885-015-1053-z PMC4343271 25886378

[14] Glynne-Jones R, Wyrwicz L, Tiret E, Brown G, Rödel C, Cervantes A (2017). Rectal cancer: ESMO clinical practice guidelines for diagnosis, treatment and follow-up. Ann Oncol.

[15] Chua YJ, Barbachano Y, Cunningham D, Oates JR, Brown G, Wotherspoon A (2010). Neoadjuvant capecitabine and oxaliplatin before chemoradiotherapy and total mesorectal excision in MRI defined poor-risk rectal cancer: a phase 2 trial. Lancet Oncol.

[16] Wang J, Fan J, Li C, Yang L, Wan J, Zhang H (2020). The impact of chemotherapy completion on the efficacy of Irinotecan in the preoperative Chemoradiotherapy of locally advanced rectal Cancer: an expanded analysis of the CinClare phase III trial. Clin Colorectal Cancer.

[17] Leichman CG, McDonough SL, Smalley SR, Billingsley KG, Lenz HJ, Beldner MA (2018). Cetuximab combined with induction Oxaliplatin and Capecitabine, followed by Neoadjuvant Chemoradiation for locally advanced rectal Cancer: SWOG 0713. Clin Colorectal Cancer.

[18] Gérard JP, Azria D, Gourgou-Bourgade S, Martel-Lafay I, Hennequin C, Etienne PL (2012). Clinical outcome of the ACCORD 12/0405 PRODIGE 2 randomized trial in rectal cancer. J Clin Oncol.

[19] Rödel C, Graeven U, Fietkau R, Hohenberger W, Hothorn T, Arnold D (2015). Oxaliplatin added to fluorouracil-based preoperative chemoradiotherapy and postoperative chemotherapy of locally advanced rectal cancer (the German CAO/ARO/AIO-04 study): final results of the multicentre, open-label, randomised, phase 3 trial. Lancet Oncol.

[20] Allegra CJ, Yothers G, O’Connell MJ, Beart RW, Wozniak TF, Pitot HC (2015). Neoadjuvant 5-FU or Capecitabine plus radiation with or without Oxaliplatin in rectal Cancer patients: a phase III randomized clinical trial. J Natl Cancer Inst.

[21] Haddad P, Miraie M, Farhan F, Fazeli MS, Alikhassi A, Maddah-Safaei A (2017). Addition of oxaliplatin to neoadjuvant radiochemotherapy in MRI-defined T3, T4 or N+ rectal cancer: a randomized clinical trial. Asia Pac J Clin Oncol..

[22] Deng Y, Chi P, Lan P, Wang L, Chen W, Cui L (2019). Neoadjuvant modified FOLFOX6 with or without radiation versus fluorouracil plus radiation for locally advanced rectal Cancer: final results of the Chinese FOWARC trial. J Clin Oncol.

[23] Tang J, Wu X, Bai Y, Gao Y, Jiang W, Kong L (2018). Long-term outcome of Oxaliplatin and Capecitabine (XELOX) concomitant with Neoadjuvant radiotherapy and extended to the resting period in high risk locally advanced rectal Cancer. J Cancer.

[24] Schmoll HJ, Haustermans K, Price TJ, Nordlinger B, Hofheinz R, Daisne JF, et al. Preoperative chemoradiotherapy and postoperative chemotherapy with capecitabine and oxaliplatin versus capecitabine alone in locally advanced rectal cancer: First results of the PETACC-6 randomized phase III trial. J Clin Oncol 2013; 31: abstr 3531.

[25] Maas M, Nelemans PJ, Valentini V (2010). Long-term outcome in patients with pathological complete response after chemoradiation for rectal cancer: a pooled analysis of individual patient data. Lancet Oncol.

[26] Duldulao MJ, Lee W, Streja L (2013). Distribution of residual cancer cells in the bowel wall after neoadjuvant chemoradiation in patients with rectal cancer. Dis Colon Rectum.

[27] Wiig JN, Larsen SG, Dueland S, Giercksky KE (2005). Clinical outcome in patients complete pathologic response (pT0) to preoperative irradiation/chemo-irradiation operated for locally advanced or locally recurrent rectal cancer. J Surg Oncol.

[28] Willett CG, Duda DG, di Tomaso E, Boucher Y, Ancukiewicz M, Sahani DV (2009). Efficacy, safety, and biomarkers of neoadjuvant bevacizumab, radiation therapy, and fluorouracil in rectal cancer: a multidisciplinary phase II study. J Clin Oncol.

[29] Velenik V, Ocvirk J, Music M, Bracko M, Anderluh F, Oblak I (2011). Neoadjuvant Capecitabine, radiotherapy, and Bevacizumab (CRAB) in locally advanced rectal Cancer: results of an open-label phase II study. Radiat Oncol.

[30] Czito BC, Bendell JC, Willett CG, Morse MA, Blobe GC, Tyler DS (2007). Bevacizumab, Oxaliplatin, and Capecitabine with radiation therapy in rectal Cancer: phase I trial results. Int J Radiat Oncol Biol Phys.

[31] Kennecke H, Berry S, Wong R, Zhou C, Tankel K, Easaw J (2012). Pre-operative Bevacizumab, Capecitabine, Oxaliplatin and radiation among patients with locally advanced or low rectal Cancer: a phase II trial. Eur J Cancer.

[32] Landry JC, Feng Y, Prabhu RS, Cohen SJ, Staley CA, Whittington R (2015). Phase II trial of preoperative radiation with concurrent Capecitabine, Oxaliplatin, and Bevacizumab followed by surgery and postoperative 5-fluorouracil, Leucovorin, Oxaliplatin (FOLFOX), and Bevacizumab in patients with locally advanced rectal Cancer: 5-year clinical outcomes ECOG-ACRIN Cancer research group E3204. Oncologist.

[33] Rödel C, Graeven U, Fietkau R, Hohenberger W, Hothorn T, Arnold D (2015). Oxaliplatin added to fl uorouracil-based preoperative chemoradiotherapy and postoperative chemotherapy of locally advanced rectal cancer (the German CAO/ARO/AIO-04 study): final results of the multicentre, open-label, randomised, phase 3 trial. Lancet Oncol.

[34] Andre T, Boni C, Navarro M, Tabernero J, Hickish T, Topham C (2009). Improved overall survival with oxaliplatin, fluorouracil, and leu- covorin as adjuvant treatment in stage II or III colon cancer in the MOSAIC trial. J Clin Oncol.

[35] Kuebler JP, Wieand HS, O’Connell MJ, Smith RE, Colangelo LH (2007). Oxaliplatin combined with weekly bolus fluorouracil and leucovorin as surgical adjuvant chemotherapy for stage II and III colon cancer: results from NSABP C-07. J Clin Oncol.

[36] Aschele C, Cionini L, Lonardi S, Pinto C, Cordio S, Rosati G (2011). Primary tumor response to preoperative chemoradiation with or without oxaliplatin in locally advanced rectal cancer: pathologic results of the STAR-01 randomized phase III trial. J Clin Oncol.

[37] O’Connell MJ, Colangelo LH, Beart RW, Petrelli NJ, Allegra CJ, Sharif S (2014). Capecitabine and oxaliplatin in the preoperative multimodality treatment of rectal cancer: surgical end points from National Surgical Adjuvant Breast and bowel project trial R-04. J Clin Oncol.

[38] Glynne-Jones R, Counsell N, Quirke P, Mortensen N, Maraveyas A, Meadows HM (2014). Chronicle: results of a randomised phase III trial in locally advanced rectal cancer after neoadjuvant chemoradiation randomizing postoperative adjuvant capecitabine plus oxaliplatin (XELOX) versus control. Ann Oncol.

[39] Hong Y, Nam B, Kim K, Kim JE, Park SJ, Park YS (2014). Oxaliplatin, fl uorouracil, and leucovorin versus fluorouracil and leucovorin as adjuvant chemotherapy for locally advanced rectal cancer after preoperative chemoradiotherapy (ADORE): an open-label, multicentre, phase 2, randomised controlled trial. Lancet Oncol.

[40] Maas M, Nelemans PJ, Valentini V, Crane CH, Capirci C, Rödel C (2015). Adjuvant chemotherapy in rectal cancer: defining subgroups who may benefit after neoadjuvant chemoradiation and resection a pooled analysis of 3313 patients. Int J Cancer.

[41] Collette L, Bosset JF, den Dulk M, Nguyen F, Mineur L, Radosevic-Jelic PM (2007). Patients with curative resection of cT3–4 rectal cancer after preoperative radiotherapy or radiochemotherapy: does anybody benefit from adjuvant fluorouracil-based chemotherapy? A trial of the European Organisation for Research and Treatment of Cancer radiation oncology group. J Clin Oncol.

[42] Douglass HO, Moertel CG, Mayer RJ, Thomas PR, Lindblad AS, Mittleman A (1986). Survival after postoperative combination treatment of rectal cancer. N Engl J Med.

[43] Fisher B, Wolmark N, Rockette H, Fisher B, Wolmark N, Rockette H, Redmond C, Deutsch M, Wickerham DL (1988). Postoperative adjuvant chemotherapy or radiation therapy for rectal cancer: results from NSABP protocol R-01. J Natl Cancer Inst.

[44] Breugom AJ, Van Gijn W, Muller EW, Berglund Å, van den Broek CBM, Fokstuen T (2015). Adjuvant Chemotherapy for Rectal Cancer Patients Treated With Preoperative (Chemo) radiotherapy and Total Mesorectal Excision: A Dutch Colorectal Cancer Group (DCCG) Randomized Phase III Trial. Ann Oncol.

