# Supplementary material for: Phase II randomized trial of capecitabine with bevacizumab and external beam radiation therapy as preoperative treatment for patients with resectable locally advanced rectal adenocarcinoma: long term results
Source: BMC Cancer. 2020 Nov 27;20:1164. doi: 10.1186/s12885-020-07661-z (PMC7694337; doi:10.1186/s12885-020-07661-z)
Supplement: Supplementary file 1 [file 12885_2020_7661_MOESM1_ESM.odt]

Supplementary Figure 1. DFS – T-downstaging and treatment arm. 

A.T No Downstaging

B-T No Downstaging

A.T-Downstaging

B-T Downstagibg

Cox regression results

| Contrast | HR pvalue | HR Estimate | CI 95% LL | CI 95% UL |
| --- | --- | --- | --- | --- |
| B-No T-Downstaging vs A- T-Downstaging | 0.0610 | 2.989 | 0.951 | 9.393 |
| B-T-Downstaging vs A- T-Downstaging | 0.5158 | 1.583 | 0.396 | 6.334 |
| A-No T-Downstaging vs A- T-Downstaging | 0.0054 | 5.092 | 1.619 | 16.017 |
