# Supplementary material for: Phase II randomized trial of capecitabine with bevacizumab and external beam radiation therapy as preoperative treatment for patients with resectable locally advanced rectal adenocarcinoma: long term results
Source: BMC Cancer. 2020 Nov 27;20:1164. doi: 10.1186/s12885-020-07661-z (PMC7694337; doi:10.1186/s12885-020-07661-z)
Supplement: Supplementary file 2 [file 12885_2020_7661_MOESM2_ESM.odt]

Supplementary Figure 2 . DFS. N-downstaging and treatment arm

A-N -Downstaging

B-N -Downstaging

A-No N -Downstaging

B-No N -Downstaging

Cox regression results

| Contrast | HR pvalue | HR Estimate | CI 95% LL | CI 95% UL |
| --- | --- | --- | --- | --- |
| B-No N-Downstaging vs A- N-Downstaging | 0.5815 | 1.654 | 0.276 | 9.900 |
| B-N-Downstaging vs A- N-Downstaging | 0.0444 | 3.628 | 1.033 | 12.744 |
| A-No N-Downstaging vs A- N-Downstaging | 0.0030 | 6.833 | 1.924 | 24.264 |
